# *Escherichia coli* phage-inducible chromosomal island aids helper phage replication and represses the locus of enterocyte effacement pathogenicity island

**DOI:** 10.1093/ismejo/wrae258

**Published:** 2025-01-05

**Authors:** Kat Pick, Lauren Stadel, Tracy L Raivio

**Affiliations:** Department of Biological Sciences, CW 405 Biological Sciences Building, University of Alberta, Edmonton, AB T6G 2E9, Canada; Department of Biological Sciences, CW 405 Biological Sciences Building, University of Alberta, Edmonton, AB T6G 2E9, Canada; Department of Biological Sciences, CW 405 Biological Sciences Building, University of Alberta, Edmonton, AB T6G 2E9, Canada

**Keywords:** phage-inducible chromosomal island, mobile genetic elements, crosstalk, lysogeny, bacterial competition, virulence, excisionase, temperate bacteriophage

## Abstract

In this study, we identify and characterize a novel phage-inducible chromosomal island (PICI) found in commensal *Escherichia coli* MP1*.* This novel element, EcCIMP1, is induced and mobilized by the temperate helper phage vB_EcoP_Kapi1. EcCIMP1 contributes to superinfection immunity against its helper phage, impacting bacterial competition outcomes. Genetic analysis of EcCIMP1 led us to uncover a putative transcriptional repressor, which silences virulence gene expression in the murine pathogen *Citrobacter rodentium.* We also found a putative excisionase encoded by EcCIMP1 which paradoxically does not promote excision of EcCIMP1 but rather supports excision of the helper phage. Another putative excisionase encoded by a presumed integrative conjugative element can also support the excision of vB_EcoP_Kapi1, demonstrating crosstalk between excisionases from multiple classes of mobile genetic elements within the same cell. Although phylogenetically distant from other characterized PICIs, EcCIMP1 and EcCIMP1-like elements are prevalent in both pathogenic and commensal isolates of *E. coli* from around the world, underscoring the importance of characterizing these abundant genetic elements.

## Graphical Abstract

**Graphical Abstract:**
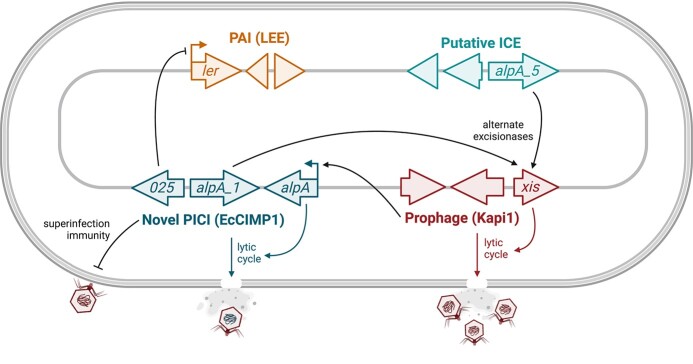

## Introduction

Mobile genetic elements (MGEs) are key drivers of bacterial evolution and diversity. By mediating horizontal gene transfer (HGT), MGEs direct the flow of genetic information through bacterial populations. Several studies have highlighted the importance of HGT in mediating bacterial adaptation within the mammalian host environment [1, 2], demonstrating that MGE-driven HGT often outweighs adaptation via mutation. Although MGEs can improve bacterial fitness via HGT, they can also be costly and even deadly to their bacterial hosts. Further complicating matters, an MGE does not exist within a vacuum and interacts both with the core genetic content of the host bacterium as well as other MGEs in the cell [3, 4]. Whether a particular bacteria–MGE interaction is positive, neutral, or detrimental to the cell is not always easily predictable, and with an ever-increasing diversity of MGEs being uncovered, much work is still needed to fully understand how MGEs impact bacterial fitness.

Temperate phages are particularly interesting MGEs from an evolutionary standpoint, as they often encode accessory genes, which can be useful to their bacterial host [5], yet they also pose a significant risk as lytic phage replication will cause cell death. Accordingly, remnants of prophages that have been mutationally inactivated over time are littered throughout bacterial genomes [6]. In addition to this, temperate phages also need to contend with their own parasites, called phage satellites. Similar to how temperate phages rely on bacteria to replicate, phage satellites rely on a helper phage for replication and mobilization between cells [7]. Phage satellites time their replication with the lytic cycle of their helper phage by sensing a phage-encoded inducing cue. After excision, circularization, and genome replication, satellite DNA is packaged into phage capsids and released from the cell by phage lysis proteins. The molecular details of each of these steps vary by satellite and can often be used to classify them into distinct satellite families: phage-inducible chromosomal islands (PICIs), phage-inducible minimalist islands (PICMIs), capsid-forming PICIs (cfPICIs), PICI-like elements (PLEs), and P4-like satellites [8, 9]. Whereas PLEs and PICMIs are only found in *Vibrionaceae*, PICIs, cfPICIs, and P4-like satellites are abundant in diverse bacterial taxa [8, 9].

Given the abundance of temperate phages in bacterial genomes [10], their potential to impact bacterial phenotypes [5], and the evolutionary forces imposed on them, we have been focused on understanding how temperate phages impact bacterial fitness. Lysogens (bacteria carrying prophages) seem to be particularly enriched in certain environments including the gastrointestinal (GI) tract, and in certain taxa including *Proteobacteria* [11]. To study these interactions, we have recently developed *Escherichia coli* MP1 lysogenized by vB_EcoP_Kapi1 (Kapi1) as a model system to study lysogeny during GI colonization [12, 13]. MP1 is a commensal strain isolated from the feces of a healthy mouse [14], and owing to its recent isolation and lack of laboratory adaptation (unlike some commonly studied K-12 strains), it has proven an excellent model system to study *E. coli* colonization of the mouse GI tract [12, 14–16]. During our initial characterization of Kapi1, we noted five additional degraded prophages present in the MP1 genome [12]. Here, we report the reclassification of one of these putative prophages as a PICI and explore its relationship with both its helper phage Kapi1, and the bacterial host MP1.

## Materials and methods

Graphical abstract was created with BioRender.com

### Bacterial strains and growth conditions

Bacteria (Supplementary Table S1) were grown in LB (10 g/l Bacto tryptone, 5 g/l Bacto yeast extract, 5 g/l NaCl, 15 g/l Bacto agar for plates) at 37°C, with shaking at 225 rpm for liquid cultures. When used, DMEM contained no phenol red (Cytiva, Vancouver Canada, CAT# SH30284.02 supplemented with sodium pyruvate to 1 mM final), and cultures were grown statically at 37°C with 5% CO_2_. Antibiotics were used at the following concentrations: ampicillin (Amp) 100 μg/ml and kanamycin (Kan) 30 μg/ml. 0.1 mM isopropyl β-D-1-thiogalactopyranoside (IPTG) was used to induce protein expression from pTrc99A. Unless otherwise specified, experiments were performed with three independent samples for each strain by picking three colonies to grow three overnight cultures for use the following day. For all CFU/ml and PFU/ml measurements, the origin of the graph represents the limit of detection.

### Cloning and strain construction

Cloning into pNLP10 [17] and pTrc99A [18] (Supplemental Table S2) was performed by restriction digest (primers listed in Supplemental Table S3). The insert for pTrc99A *alpA_3* was synthesized by Integrated DNA Technologies (Coralville IA, US), then subcloned out of pIDTSmart into pTrc99A. All pRE112 [19] inserts were generated by OE-PCR [20]. All plasmid inserts were confirmed by Sanger sequencing by the University of Alberta Molecular Biology Service Unit.

MP13 ∆*xis* was constructed by Lambda Red recombination using the pKD13 template plasmid [21], followed by P1 transduction [22, 23] of the sequenced mutation into a fresh MP13 background and removal of the kanamycin cassette with FLP-mediated recombination [21]. MP1 ∆*cpxR::kan* was constructed by P1 transduction [22, 23], using JW3883 as the donor strain [24]. All other chromosomal deletions and insertions were performed by allelic exchange [25]. EcCIMP1*::kan* was generated by inserting the *kan* cassette (including 364 bp upstream from the start codon) from pKD13 [21] in between EcCIMP1_022 and EcCIMP1_023, as to not disrupt any potential promoters. Deletion of Kapi1 was performed by streaking out a lysogenic strain on LB, followed by replica plating onto a lawn of KP7, where nonlysogens are identified by the absence of a zone of lysis in the KP7 lawn around the colony [12] and confirmed by polymerase chain reaction (PCR).

### Genome annotation

The EcCIMP1 genome was annotated as described in [12], with the addition of InterProScan [26, 27] at EMBL-EBI [28] and DeepTMHMM [29] to aid in functional assignment [30].

### Excision PCRs

PCRs to detect excision of EcCMP1 were performed using Taq polymerase (Invitrogen, Waltham MA, USA) according to the manufacturer’s directions, using 2.5 μl overnight culture per 25 μl reaction as template. Initial denaturation was performed at 95°C for 3 min followed by 30 cycles of denaturation at 95°C for 30 s, annealing for 30 s (annealing temperatures for each primer pair are listed in Supplemental Table S3), and extend at 72°C for 4.5 min, with a final extension at 72°C for 10 min. 10 μl of each PCR reaction was separated on a 1% agarose gel and stained with GelGreen (Biotium, Fremont CA, USA). PCRs to detect excision of Kapi1 were performed as above, using 2.5 ul supernatant as template, 90 s/kb extension time, and the agarose gel was stained with ethidium bromide. PCR products were sequenced (Sanger) by the University of Alberta Molecular Biology Service Unit.

### Transduction

Kapi1 lysogens carrying EcCIMP1::*kan* were subcultured into 2 ml LB containing 5 mM CaCl_2_ and 0.2% glucose for 30 min. 0.5 ng/μl mitomycin C was added, and cultures were grown for 2–3 h. 40 μl chloroform was added and cultures were vortexed to lyse cells, then cell debris was pelleted at 15000 rpm for 1 min. The resulting lysate was transferred into a fresh tube, where another 40 μl chloroform was added and was then titered to ensure all lysates contained approximately equal Kapi1 titers by spotting 5 μl serially diluted lysate onto a solidified top agar plate (LB agar overlaid with 3.5 ml soft agar (LB 0.75% agar) mixed with 50 μl KP7 overnight culture) and incubated overnight at 30°C.

A 5 ml overnight culture of KP7 ∆EcCIMP1 was pelleted at 1500×*g* for 10 min, then resuspended in 2.5 ml sterile distilled water containing 10 mM MgSO_4_ and 5 mM CaCl_2_. 100 μl of these cells were mixed with 100 μl of the above lysate and incubated at 30°C for 30 min (static). As a control, 100 μl of the recipient strain was mixed with 100 μl LB to confirm that any subsequent growth on LB + Kan agar was not due to spontaneous resistance. 1 ml LB containing 10 mM Na-citrate was added, and cells were incubated at 37°C for 30 min (static). Cells were pelleted at 1500×*g* for 10 min, resuspended in 200 μl 1 M Na-citrate, serially diluted in 1 M Na-citrate, spread onto LB + Kan agar and incubated overnight to enumerate transductants/ml.

### Luminescent reporter assays

Overnight cultures were subcultured into 2 m LB + Kan (+Amp for strains additionally carrying pTrc99a) for 2 h. For the *alpA-lux* experiment, induction was performed by the addition of 0.25 ng/ul mitC. For *LEE1-lux* experiments, cells were pelleted at 4000 rpm for 5 min, then resuspended in either fresh LB + Amp + Kan, LB + Amp + Kan + IPTG, DMEM+Amp + Kan, or DMEM+Amp + Kan + IPTG. Cultures were then returned to their respective incubators, and at the indicated time points (setting the induction or centrifugation step as t0), 100 μl aliquots were transferred to a black 96-well plate. Luminescence (counts per second, CPS) and optical density at 600 nm (OD_600_) were immediately measured using the PerkinElmer (Waltham MA, US) Victor X3 2020 multilabel plate reader.

### Bacterial competition assays

Bacterial competition assays were performed as described in [12], except that OD_600_ adjustments were performed in LB, and strains were subcultured into LB only. Briefly, we cocultured MP1 derivatives chromosomally tagged with either *gfpmut3.1* or *mcherry* [14]. Every 24 h, the number of green- and red-fluorescent colonies is enumerated by plate count, and the coculture is diluted into fresh LB for another 24 h. The competitive index (CI) is calculated as ((*gfp* CFU/ml/*mcherry* CFU/ml)/(*gfp* CFU/ml input/*mcherry* CFU/ml input)).

### Prophage induction and titering

Overnight cultures were subcultured into 2 ml LB (+Amp for strains carrying pTrc99a) for 2 h, then mitomycin C was added to 0.5 ng/μl, and cultures were incubated for an additional 2 h. Cells were pelleted at 15000 rpm for 5 min; the supernatant was serially diluted in PBS and titered as above. For complementation experiments, IPTG was added 1 h before the addition of mitomycin C.

### Bacterial genome sequencing and variant calling

Genomic DNA was extracted from 500 μl of overnight cultures of MP13 ∆*xis* and MP13 ∆*int* using the BioSearch (Teddington, UK) MasterPure Complete DNA and RNA Purification Kit following the manufacturer’s protocol, except DNA was resuspended in nuclease-free water rather than TE buffer. Sequencing was performed by SeqCenter (Pittsburgh, PA) using the Illumina (San Diego, CA, USA) Library Prep Kit and custom IDT (Coralville, IA, USA) 10 bp unique dual indices with a target insert size of 280 bp on an Illumina NovaSeq X Plus. Demultiplexing, quality control, and adapter trimming were performed with bcl-convert v4.2.4.

Read trimming and quality control were performed using fastp [31]. Variant calling was performed with breseq [32], using the trimmed reads as input, and the WT MP1 reference genome (accession CP139039-40). Unmapped reads from breseq were also assembled with SPAdes [33] via Galaxy [34] to ensure that no new sequences were acquired by the ∆*xis* or ∆*int* mutants. Unmapped reads from both strains corresponded to the expected *tetRA-gfp* cassette inserted into MP13 [14].

## Results

### Identification and characterization of novel PICI, EcCIMP1

**Figure 1:**
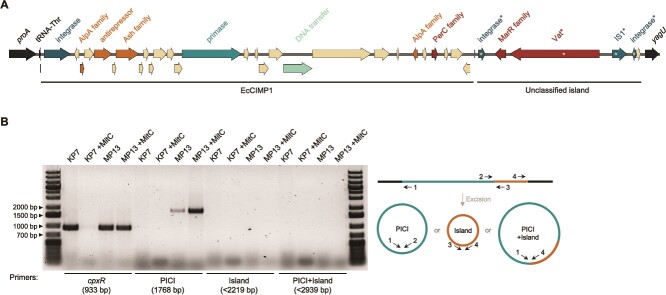
EcCIMP1 is a novel phage-inducible chromosomal island. (A) Genetic map of the *E. coli* MP1 chromosome where EcCIMP1 and an adjacent unclassified genomic island are integrated (accession CP139039, 3 585 854–3 557 883 bp). EcCIMP1 CDS were manually annotated and are color-coded based on predicted function: chromosomal genes flanking the islands are shown in black (*proA, yagU*), genes involved in DNA movement in dark blue (integrase, IS1), DNA primase in blue, DNA transfer in light blue, transcriptional regulation in orange (AlpA, antirepressor), and virulence in red (PerC, MarR, Vat). Asterisks (^*^) indicate pseudogenes. Visualization was generated with SnapGene software (www.snapgene.com). (B) PCR on cultures of MP1 nonlysogens (KP7) and Kapi1 lysogens (MP13). 0.125 μg/ml mitomycin C was added to bacterial cultures where indicated (+MitC) to induce Kapi1 into the lytic cycle. Primers used in each PCR are shown in the schematic to the right. Primers for chromosomal *cpxR* serve as a positive amplification control for each sample. Experiment was repeated twice, with one representative shown.

Upon closer inspection of the Incomplete_1 prophage region previously identified in MP1 [12], we realized that this may not be a degraded prophage, but instead two smaller genetic elements; a PICI and an unclassified genomic island (Fig. 1A). Annotation of the putative PICI revealed coding sequences (CDS) more characteristic of PICIs than of bacteriophages, including an integrase, AlpA and PerC family transcriptional regulators, and DNA primase (Fig. 1A). The genetic organization and size of this putative PICI are consistent with characterized *E. coli* PICIs [8, 35, 36], and no phage-like structural or lysis genes were identified. The adjacent island is ~7 kbp, encoding a MarR family transcriptional regulator and four pseudogenes (Fig. 1A).

To confirm that this region does contain a PICI, we performed PCR with primers for PICI excision and circularization (targeting the PICI *attP*) on cultures of MP1 either carrying (MP13) or lacking (KP7) the Kapi1 prophage. MP13 is a derivative of MP1 with a chromosomal *gfpmut3.1* locus under the control of *tetRA* [14] and is a natural Kapi1 lysogen [12]. KP7 is similarly tagged with *mcherry* [14], but has lost the Kapi1 prophage [12]. We included additional primers to determine if the adjacent island is capable of excision and circularization but did not observe bands under any conditions tested (Fig. 1B). These PCRs confirmed our classification of the PICI, as circularization is only detected when the helper phage Kapi1 is present, and further increases when Kapi1 is induced into the lytic cycle with mitomycin C (Fig. 1B). Sanger sequencing of the resulting PCR product confirmed the exact boundaries of the PICI, which is 19 963 bp in length, corresponding to base pairs 3 565 708-3 585 670 in the MP1 genome (accession CP139039). The PICI and adjacent island are integrated into tRNA-Thr in between *proA* and *yagU.* We identified two direct repeats (TCCTATTATC) flanking the PICI that we hypothesize represent the core *att* sequence, which is identical to the predicted *att* site for PICI CnCIND14b of *Cedecea neteri* [35]. In our search of the literature and sequence databases, we did not find any mention of this PICI and have therefore named this novel PICI EcCIMP1, following the standard nomenclature (*E. coli*chromosomal island in strain MP1). We have deposited the nucleotide sequence of EcCIMP1 including our annotations at Genbank accession OR778291.

**Figure 2:**
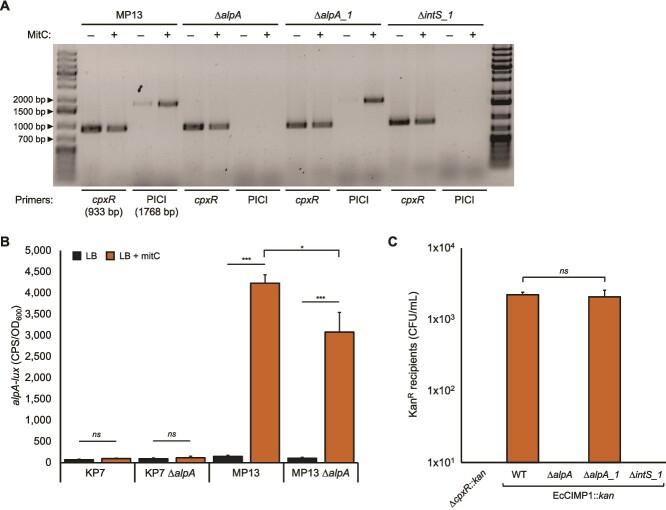
EcCIMP1 encodes two AlpA family transcriptional regulators with differing functions in the PICI cycle. (A) PCR on overnight cultures of Kapi1 lysogens. 0.125 μg/ml mitomycin C was added to bacterial cultures where indicated (+MitC) to induce Kapi1 into the lytic cycle. Primers are the same used in Fig. 1B. Experiment was repeated twice, with one representative shown. (B) Luminescent reporter assay of EcCIMP1 *alpA-lux* expression in MP1 nonlysogens (KP7) or Kapi1 lysogens (MP13). Luminescence (measured in counts per second, CPS, and standardized to bacterial growth, OD_600_) was monitored for 2 h after addition of 0.25 ng/μl mitomycin C (+ mitC), with data from the 90-min time point shown. Experiment was repeated three times, with one representative shown. The mean of three independent samples is reported, with the standard deviation shown as error bars. Statistical significance was determined using a *t*-test (^*^P ≤ .05, ^***^P ≤ .001). (C) Transduction of EcCIMP1::*kan* from Kapi1 lysogens into ∆EcCIMP1 ∆Kapi1 recipient. ∆*cpxR::kan* was used as a control for generalized transduction. Each experiment was performed with one biological replicate, and the experiment was repeated three times. The mean is shown, with the standard deviation as error bars. Statistical significance was determined using a *t*-test.

During annotation of EcCIMP1, we noted two AlpA family transcriptional regulators (Fig. 1A), whereas typical *E. coli* PICIs only encode one. In Gram-negative PICIs AlpA is the master regulator of the PICI cycle; when the helper phage is induced into its lytic cycle *alpA* expression increases and is ultimately required for proper expression of PICI lytic genes and mobilization of the element [35]. EcCIMP1 encodes one AlpA family transcriptional regulator in the conserved location downstream of the integrase (locus_tag EcCIMP1_003, *alpA*), but the second copy (EcCIMP1_023, *alpA_1*) is in an unusual location at the opposite end of the genome and is encoded on the opposite strand from most other EcCIMP1 CDS (Fig. 1A). PCR for EcCIMP1 excision and circularization in Kapi1 lysogens showed that the canonical *alpA* is required for EcCIMP1 excision and circularization, whereas the unusual *alpA_1* is dispensable (Fig. 2A), suggesting that *alpA* may be the master regulator of EcCIMP1. As a control, we also included a ∆*intS_1* (EcCIMP1_001) mutant, as the PICI integrase is required for excision (Fig. 2A).

In other Gram-negative PICIs, AlpA is expressed upon induction of its cognate helper phage and acts as a positive autoregulator, boosting its own expression [35]. To determine if our putative master regulator *alpA* has the same expression profile, we generated a luminescent reporter plasmid [17] to monitor activity of the *alpA* promoter region (−449 bp to +50 bp)*.* EcCIMP1 *alpA-lux* expression is strongly induced in Kapi1 lysogens when the helper phage is induced with mitomycin C but not in a mitomycin C-treated nonlysogen (Fig. 2B), confirming that *alpA* expression is specifically induced by the helper phage and not by DNA damage itself. In an ∆*alpA* mutant*,* we still see mitomycin C-dependent upregulation of the reporter plasmid *alpA-lux*, but it does not reach the maximal level seen in the WT lysogen, demonstrating that AlpA likely acts as a positive autoregulator in EcCIMP1 (Fig. 2B), as has been observed for other PICIs [35].

Although we have evidence that EcCIMP1 excises from the bacterial chromosome and circularizes, we wanted to verify that, like other PICIs, EcCIMP1 DNA is specifically packaged inside Kapi1 particles and can be transferred into naïve cells. EcCIMP1::*kan* is successfully transduced from Kapi1 lysogens into ∆EcCIMP1 ∆Kapi1 recipients (Fig. 2C). A control experiment using a Kapi1 lysogen with a chromosomal ∆*cpxR*::*kan* tag as the donor strain (Fig. 2C) confirmed that Kapi1 is not a generalized transducing phage, so EcCIMP1::*kan* transfer occurs through a specific phage-PICI interaction. Consistent with our PCR results (Fig. 2A), EcCIMP1::*kan* was not mobilized when the donor strain had either the master regulator *alpA* or the integrase *intS_1* deleted, whereas an ∆*alpA_1* mutant retained mobility (Fig. 2C). We thus conclude that EcCIMP1 is a fully functional PICI that is mobilized by its helper phage Kapi1, and that *alpA* is the master regulator of EcCIMP1, whereas the unusual *alpA_1* locus has a yet-to-be-determined function.

### EcCIMP1 impacts bacterial fitness and virulence gene expression

Many MGEs (including PICIs) carry accessory genes that impact the physiology of their bacterial host but do not contribute directly to the lifecycle of the MGE itself. We noted two CDS encoded on EcCIMP1, which may have accessory functions. The first, EcCIMP1_002, shows high amino acid sequence similarity to excinuclease ABC subunit B (*uvrB*) of *E. coli* and *Salmonella enterica.* However, overexpression of EcCIMP1_002 did not rescue *E. coli* BW25113 ∆*uvrB::kan* mutants from UV exposure (Supplemental Fig. S1), suggesting it does not act as an excinuclease. Upon closer investigation of our BLASTp [37] results, we found that all of the UvrB protein records were annotated based on similarity to a single suppressed hypothetical protein record (accession WP_001304884.1) which is no longer annotated on any genome. Although we cannot conclusively rule out that these sequences could have UvrB-like functions, the weak annotation evidence and the fact that they are only 10% of the size of *E. coli* chromosomal UvrB raise questions regarding their annotation as UvrB. This is an important example of how easily annotation errors can be propagated through public databases.

**Figure 3:**
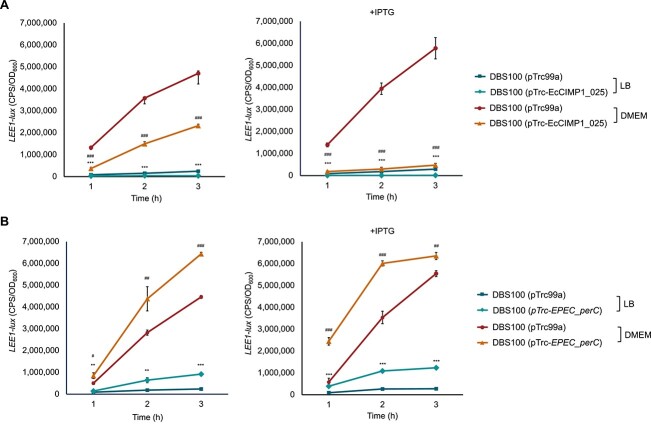
EcCIMP1 encodes a putative transcriptional repressor which silences virulence gene expression in *Citrobacter rodentium*. Luminescent reporter assays of *LEE1-lux* expression (measured in counts per second, CPS, and standardized to bacterial growth, OD_600_) in *C. rodentium* DBS100 carrying plasmids expressing (A) EcCIMP1_025, (B) *EPEC_perC*, or empty vector controls. Experiments were repeated twice, with one representative shown. The mean of three independent samples is reported, with the standard deviation shown as error bars. Statistical significance was determined using a *t*-test comparing strains carrying pTrc-EcCIMP1_025 or *EPEC_perC* to the empty vector control for each media type at each time point. Where indicated, IPTG was added to induce protein expression from pTrc99a. ^*^ is used to denote statistical significance for LB samples, # is used for statistical significance in DMEM (^*^ or #*P* < .05, ^**^ or ##*P* < .01, ^***^ or ### *P* ≤ .001).

The second putative accessory gene encoded by EcCIMP1 is EcCIMP1_025, which shows amino acid sequence similarity and conserved domains [38, 39] with PerC family transcriptional regulators. PerC is a plasmid-encoded transcriptional activator of the locus of enterocyte effacement (LEE) pathogenicity island (PAI) in enteropathogenic *E. coli* (EPEC) [40, 41]. Expression of EcCIMP1_025 in *Citrobacter rodentium* DBS100 (a LEE-containing organism used as a murine model for EPEC [42]) actually silences *LEE1*-*lux* expression, in both noninducing (LB) and virulence-inducing conditions (DMEM) (Fig. 3A). As a control, we repeated this assay with *perC* from EPEC cloned into DBS100, confirming that *perC* activates virulence gene expression as expected (Fig. 3B). In both cases, a dose-dependent response is observed when EcCIMP1_025 or *perC* are overexpressed by the addition of IPTG (Fig. 3). Although bioinformatic analyses support functional annotation of EcCIMP1_025 as a PerC family transcriptional activator, our experimental evidence suggests that it instead functions as a repressor.

**Figure 4:**
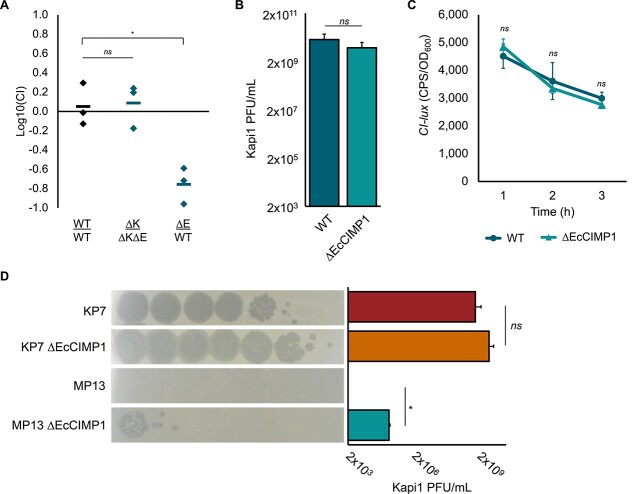
EcCIMP1 contributes to Kapi1 superinfection immunity. (A) Bacterial competition outcomes after 48 h coculture in LB. WT MP1 carries both Kapi1 and EcCIMP1, ∆K indicates deletion of the Kapi1 prophage, and ∆E indicates deletion of EcCIMP1. The strain designated as the numerator is tagged with *gfp*, and the denominator is tagged with *mcherry*. The CI was calculated as ((*gfp* CFU/ml/*mcherry* CFU/ml)/(*gfp* CFU/ml input/*mcherry* CFU/ml input)). Experiment was repeated twice, with one representative shown, where independent samples are plotted as diamonds, and the mean value is shown as a horizontal bar. Statistical significance was determined with a *t*-test comparing each CI to the control (WT/WT). (B) Titer of Kapi1 particles released following induction of Kapi1 lysogens with 0.5 ng/μl mitomycin C. Experiment was repeated twice, with one representative shown. The mean of three independent samples is reported, with the standard deviation shown as error bars. Statistical significance was determined with a *t*-test. (C) Luminescent reporter assay monitoring Kapi1 *CI-lux* expression (measured in counts per second, CPS, and standardized to bacterial growth, OD_600_) in Kapi1 lysogens. Experiment was repeated twice, with one representative shown. The mean of three independent samples is reported, with the standard deviation shown as error bars. Statistical significance was determined using a *t*-test comparing WT and ∆EcCIMP1 at each time point. (D) Susceptibility to infection by Kapi1. Each experiment was performed with one biological replicate, and the experiment was repeated twice, with the images from one representative shown. The mean of both experiments is plotted, with the standard deviation shown as error bars. Statistical significance was determined using a *t*-test comparing WT and ∆EcCIMP1 for each strain background (^*^P ≤ .05).

Given the presence of at least one functional accessory gene encoded by EcCIMP1, we investigated whether EcCIMP1 has any broader impact on bacterial fitness. Deletion of EcCIMP1 does not impact the growth of MP1 in either laboratory (LB) or GI-mimicking media (simulated gastric (SGF) [12, 43], intestinal (SIF) [12, 43], colonic (SCFM) [44, 45] fluids) (Supplemental Fig. S2). However, competition assays performed by coculturing tagged WT and ∆EcCIMP1 Kapi1 lysogens demonstrate that EcCIMP1 does provide a fitness benefit in coculture (Fig. 4A). EcCIMP1 does not impact competitive outcomes in cocultures of WT and ∆EcCIMP1 nonlysogens (Fig. 4A), suggesting that the fitness benefit afforded by EcCIMP1 is somehow related to Kapi1. In our system, competition outcomes seem to be primarily driven by induced prophages from lysogens killing susceptible competitor strains (Supplemental Fig. S3A), as has been hypothesized for other prophages [46, 47]. We hypothesized that EcCIMP1 might be altering Kapi1 infection dynamics in coculture but found that Kapi1 titers liberated from induced lysogens (Fig. 4B) and expression of the Kapi1 master regulator *CI-lux* (Fig. 4C) were not altered by EcCIMP1. However, we found that EcCIMP1 contributes to superinfection immunity of Kapi1 lysogens against subsequent infections by Kapi1 (Fig. 4D). Further competition assays confirmed that the fitness benefit from EcCIMP1 is only relevant in the lysogenic strain background and only when in competition with another lysogen (Supplemental Fig. S3B). Together these results suggest that EcCIMP1-mediated superinfection immunity can promote bacterial fitness in mixed communities where lysogens are at risk of superinfection.

### EcCIMP1 is present in globally distributed *E. coli* strains and is phylogenetically distinct from other PICIs

**Figure 5:**
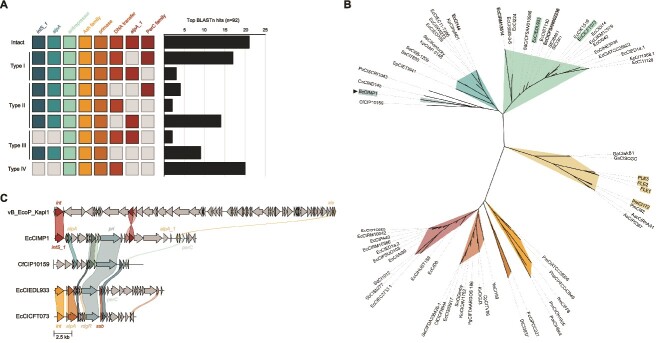
Distribution and phylogeny of EcCIMP1 reveals a unique yet widespread PICI. (A) Number of strains in the NCBI core_nt database [48] which carry EcCIMP1 or EcCIMP1-like regions based on BLASTn analysis [37]. Squares indicate the presence (color) or absence (gray) of each annotated EcCIMP1 gene. EcCIMP1-like sequences are clustered into four main types, based on the number of genes they are missing relative to EcCIMP1 (type I: one gene missing, type II: two genes missing, type III: three genes missing, type IV: four genes missing). (B) Unrooted phylogenetic tree based on nucleotide sequences. PICI and PLE names highlighted in color have been experimentally characterized, whereas all others have only been bioinformatically predicted. Visualization was generated with iTOL [49]. (C) Clinker [50] alignment of EcCIMP1 CDS amino acid sequences with the most similar virus (Kapi1), the most similar PICI (CfCIP10159), and the two experimentally characterized PICIs from *Escherichia coli* (EcCIEDL933, EcCICFT073). Genes are color-coded according to similarity groups determined by clinker.

Given that EcCIMP1 can impact host gene expression and fitness (Figs. 3-4), we used BLASTn [37] to search the NCBI core_nt database [48] to determine if EcCIMP1 is found in any other strains besides MP1. We found 21 strains of *E. coli* in that carry EcCIMP1 and many more encoding EcCIMP1-like elements (Fig. 5A). The strains carrying intact EcCIMP1 show an approximately equal distribution of commensal and pathogenic isolates, with a broad geographic distribution (Supplemental Table S4). In the strains with only partial similarity to EcCIMP1, we observed strong conservation in the antirepressor, Ash family protein, and DNA primase, whereas the presence of the integrase and AlpA and PerC family transcriptional regulators was more variable (Fig. 5A). Only two of the 21 EcCIMP1-containing strains also carry a Kapi1-like prophage (Supplemental Fig. S4), suggesting that EcCIMP1 may be able to utilize diverse helper phages for its mobilization.

To perform a phylogenetic analysis of EcCIMP1, we compiled the nucleotide sequences of previously identified PICIs found in Gram-negative bacteria [35, 36] and PICI-like elements (PLEs) from *Vibrio cholerae* [51, 52] (Supplemental Table S5). EcCIMP1 shows limited sequence similarity to all PICIs analysed, forming an outgroup with putative PICIs from *Pectobacterium carotovora* subsp*. atroseptica, C. neteri*, and *Citrobacter freundii* [35], and clustering away from *E. coli* and *Shigella* spp. PICIs [35, 36] (Fig. 5B). To visualize the similarities and differences of EcCIMP1 to other PICIs at the level of CDS, we used clinker [50] to align amino acid sequences of EcCIMP1 with its most closely related PICI CfCIP10159, two experimentally characterized *E. coli* PICIs EcCICFT073 and EcCIEDL933, and its helper phage Kapi1. This alignment revealed similarities among the PICIs in the previously discussed transcriptional regulators AlpA and PerC, some hypothetical proteins, and the DNA primase (Fig. 5C). Four CDS on EcCIMP1 share sequence similarity with its own helper phage Kapi1; an integrase, excisionase, and two hypothetical proteins that may be involved in virion morphogenesis (Fig. 5C).

### Excisionase crosstalk between diverse MGEs in MP1

**Figure 6:**
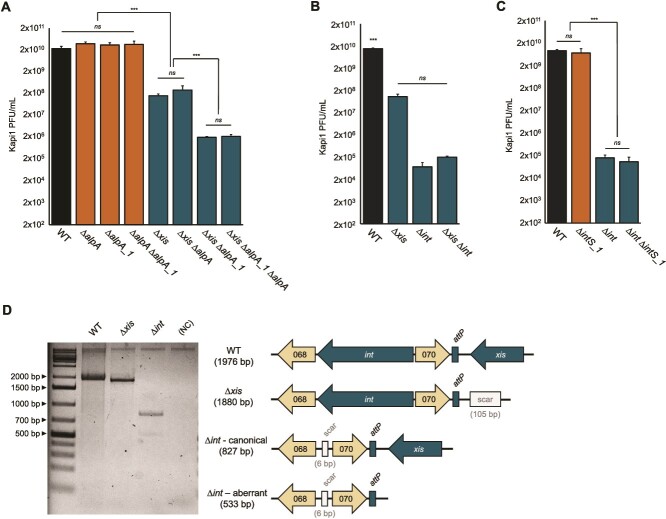
Deletion of Kapi1 excisionase or integrase does not abolish prophage excision, and EcCIMP1 *alpA_1* contributes to Kapi1 lytic replication. (A, B, C) Kapi1 lysogens were grown to mid-log phase, induced with mitomycin C, and the resulting supernatant titered by plaque assay. EcCIMP1 mutants are plotted in orange (∆*alpA*, ∆*alpA_1*, ∆*alpA* ∆*alpA_1*, ∆*intS_1*), and Kapi1 and Kapi1 EcCIMP1 double mutants are plotted in blue (all remaining mutants). Experiments were repeated twice, with one representative shown. The mean of three independent samples is plotted, with the standard deviation shown as error bars. Statistical significance was determined using *t* tests (^***^P ≤ .001) (A), or one-way ANOVA (^***^P ≤ .001) (B, C). (D) PCR and sanger sequencing of phage lysates from (B) using primers targeting Kapi1 *attP*. NC = negative control using no template in the PCR reaction.

We previously showed that EcCIMP1 *alpA_1* was not essential to the PICI cycle (Fig. 2) and was therefore unlikely to be the true AlpA master regulator. The fact that AlpA_1 is similar to Kapi1 excisionase Xis (Fig. 5C) led us to hypothesize that *alpA_1* might function as an excisionase, but rather than mediating excision of EcCIMP1, it could contribute to excision of its helper phage, Kapi1. We generated a series of deletion mutants lacking Kapi1 excisionase *xis* (accession MT813197.1, locus_tag Kapi1_001) and the two genes on EcCIMP1 with sequence similarity to *xis*; *alpA* and *alpA_1*. We induced each of these mutant lysogens with mitomycin C and measured the impact on Kapi1 excision and lytic replication by plaque assay. Although deletion of *alpA* and *alpA_1* did not impact Kapi1 titers in a WT lysogen, deletion of *alpA_1* significantly reduced Kapi1 titers in a ∆*xis* background (Fig. 6A), suggesting that *alpA_1* may be able to stand-in for Kapi1 native excisionase, although at a reduced efficiency relative to WT. We repeated the same process to test whether the EcCIMP1 integrase *intS_1* can functionally complement Kapi1 integrase *int* (Kapi1_069), as they also share amino acid sequence similarity*.* However, deletion of EcCIMP1 integrase did not impact Kapi1 titers in either a WT or ∆*int* background (Fig. 6B).

During these assays, we noted that Kapi1 ∆*xis* and ∆*int* mutants were still able to excise and replicate in response to mitomycin C induction, and even a double ∆*xis* ∆*int* mutant still produces viable plaque-forming particles (Fig. 6C). Although Kapi1 is the only active prophage in MP1 [12], we confirmed that the observed plaques were indeed Kapi1 ∆*xis* or ∆*int* by PCR with primers targeting Kapi1 *attP* on lysates (Fig. 6D), and the resulting plaques (data not shown). Sanger sequencing of the *attP* PCR products confirmed canonical prophage excision in both the ∆*xis* and ∆*int* mutants and a minor aberrant excision product for the ∆*int* mutant (Fig. 6D). Because the aberrant excision band is very faint, and we never observed it on PCRs of individual ∆*int* plaques (only in lysates), it is likely a very rare event. Finally, we performed high-throughput sequencing on bacterial genomic DNA from the ∆*xis* and ∆*int* mutants to rule out the presence of off-site mutations that could allow Kapi1 excision to proceed in the absence of its excisionase and integrase. Variant calling confirmed the expected ∆*xis* and ∆*int* mutations, and we only observed one off-site mutation in *galU* (L34Q) in the ∆*xis* strain. *galU* is a UTP—glucose-1-phosphate uridylyltransferase involved in LPS biosynthesis [53], so it is very unlikely that this single amino acid change would impact DNA recombination or prophage excision.

**Figure 7:**
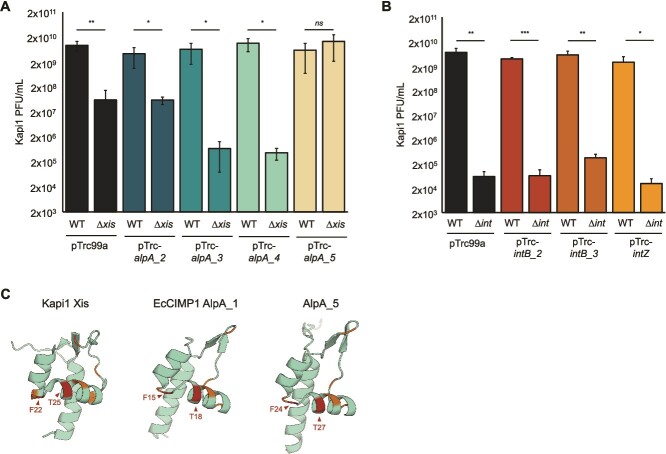
MGE-encoded excisionases in MP1 can inhibit or promote Kapi1 excisive recombination. (A, B) Kapi1 plaque production was measured from Kapi1 lysogens carrying pTrc99a expression vectors induced with 0.1 mM IPTG prior to mitomycin C prophage induction. Experiments were repeated twice, with one representative shown. The mean of three independent samples is plotted, with the standard deviation shown as error bars. (A) Statistical significance was determined for each vector with a *t*-test comparing the WT and ∆*xis* strains (^*^ one-tailed *P* < .05, ^**^ one-tailed *P* < .01). (B) Statistical significance was determined for each vector with a *t*-test comparing the WT and ∆*int* strains (^*^ one-tailed *P* < .05, ^**^ one-tailed *P* < .01, ^***^ one-tailed *P* < .001). (C) Experimental structure for a protein with 100% sequence identity to Kapi1 Xis (PDB 1Z4H), and Alphafold predicted structures for AlpA_1 (Uniprot D7XGK5) and AlpA_5 (Uniprot A0A780RR38). Residues conserved between all Kapi1 Xis-like proteins are highlighted in orange, whereas residues uniquely conserved between only Xis, AlpA_1, and AlpA_5 are highlighted in red and indicated with arrows. Protein structures were visualized with the PyMOL Molecular Graphics System (v2.0 Schrödinger, LLC).

Because we observed that EcCIMP1 *alpA_1* can support Kapi1 excision in the absence of its native excisionase (Fig. 6A), we hypothesized that there could be even more excisionases and integrases in MP1 that might explain how Kapi1 ∆*xis* and ∆*int* mutants can still perform canonical prophage excision. Using BLASTp [37], we found four excisionases and three integrases in the MP1 genome ranging from 14 to 36% normalized amino acid % identity (% identity ^*^ % query coverage) to Kapi1 Xis and Int (Supplemental Table S6, S7). All the excisionases are annotated as AlpA family transcriptional regulators, suggesting that this protein family description may be too general, since there may be subfamilies with differing functions as we have demonstrated with the transcriptional regulator *alpA* and excisionase *alpA_1* (Figs 2 and 6A). All seven integrases and excisionases are encoded on prophages predicted by PHASTER [54, 55], or on genomic islands predicted by GIPSy [56] (Supplemental Tables S6 and S7). We cloned each gene of interest into an expression vector and again monitored plaque formation from induced Kapi1 ∆*xis* or *∆int* lysogens to test for complementation. Overexpression of *alpA_5* completely rescued the replication defect of Kapi1 ∆*xis* mutants (Fig. 7A), suggesting that it is a functional excisionase. Conversely, overexpression of *alpA_3* and *alpA_4* further inhibited Kapi1 replication in the ∆*xis* mutant (Fig. 7A). None of the tested integrases had any impact on Kapi1 replication in either the WT or ∆*int* background (Fig. 7B). Using the AlphaFold Protein Structure Database [57, 58], we then compared the predicted structures for each of the excisionases, noting a high degree of structural similarity despite their relatively low amino acid similarity. We then performed a MUSCLE [59] alignment via EMBL-EBI [28] (Supplemental Fig. S5); although all seven putative excisionases share some conserved residues (highlighted in orange, Fig. 7C), we identified two residues that were uniquely conserved among only the proteins that successfully complemented Kapi1 Xis (highlighted in red with arrows, Fig. 7C). Comparing the location of these conserved residues with the topology of the Lambda Xis–DNA complex (PDB 1RH6) [60, 61], we speculate that these residues may lie within the DNA recognition and binding region of the protein [62].

## Discussion

Here, we identify a novel PICI found in commensal *E. coli* MP1, which relies on its helper phage Kapi1 for mobilization. Although EcCIMP1 is phylogenetically distant from other Gram-negative PICIs, it is present in at least 22 strains of *E. coli* with broad geographical distribution (Supplemental Table S4). EcCIMP1 shares some core genes with other characterized PICIs (integrase, AlpA family transcriptional regulator, and DNA primase/helicase) [8, 35, 36] (Fig. 1A), but is missing identifiable capsid morphogenesis or terminase genes, and generally lacks similarity in the packaging module (downstream of DNA primase) to PICIs with well-characterized packaging mechanisms [36, 63] (Fig. 5C). Using the scoring scheme developed by de Sousa et al. [8], EcCIMP1 would therefore be classified as a Type C #var01 PICI, which are abundant in bacterial genomes but cannot be confidently annotated as they are missing key modules suggesting they may be defective, or another type of genetic element altogether. We experimentally verified that EcCIMP1 is a functional PICI as it is capable of excision and circularization only when its helper phage Kapi1 is induced into the lytic cycle (Fig. 1B) and can be transferred to naïve strains by Kapi1 (Fig. 2C), suggesting its DNA is packaged in Kapi1 capsids. Hypothetical proteins EcCIMP1_017 through _019 are promising candidates for being involved in EcCIMP1 DNA packaging, as they are encoded in the typical location downstream of the DNA primase (Fig. 1A) [8, 35, 36] and have some degree of similarity to DNA transfer proteins and DUF2824 domain-containing proteins which may be involved in phage head morphogenesis [39]. These CDS also share sequence similarity with hypothetical proteins encoded by Kapi1 (Fig. 5C), which have some similarity to DNA transfer proteins and phrog_651 conserved domain-containing proteins involved in phage head morphogenesis [64]. However, due to the low rate of transduction of EcCIMP1 by Kapi1 (Fig. 2C), it is difficult to characterize what mechanism EcCIMP1 may use for packaging.

In our previous work [12], we asserted that the region corresponding to EcCIMP1 and the adjacent genomic island were likely a defective prophage (called Incomplete_1), as we could not detect Incomplete_1 DNA by PCR of DNase-treated culture supernatants. However, due to the extremely low transduction rate for EcCIMP1::*kan* (Fig. 2C), we believe that our previous PCR results were a false negative. Using the number of observed transductants, we estimate that 4.8 × 10^3^ particles/ml contain EcCIMP1::*kan*. Given that 5 μl of culture supernatant was used for PCR in our previous work [12], we could expect approximately 24 EcCIMP1-containing particles in each PCR reaction, which would provide 0.0005 pg EcCIMP1 DNA template per reaction, far below the minimum (1 pg) required for Taq polymerase (Invitrogen).

We identified a PerC family transcriptional regulator encoded by EcCIMP1, which are virulence factors commonly found in PICIs in Gram-negative bacteria [35, 36]. Whereas PerC is normally an activator of the LEE pathogenicity island in EPEC [40, 41], the PerC-like gene carried by EcCIMP1 (EcCIMP1_025) has the opposite effect, strongly silencing LEE expression in the murine pathogen *C. rodentium* (Fig. 3A). Although PICIs EcCIEDL933 and EcCICFT073 also encode PerC-like genes with ~40% amino acid identity to EcCIMP1_025, they were previously shown to have no impact on the expression of *LEE1*-*lacZ* promoter fusions from EPEC or EHEC [65]. During annotation of EcCIMP1_025, we noted a few ANR family transcriptional regulators further down in our BLASTp [37] and conserved domain [38] search results. Given our experimental evidence that EcCIMP1_025 represses LEE, we hypothesize that it may instead be an ANR family repressor. The AraC Negative Regulator (ANR) family is widespread in Gram-negative pathogens and reduces virulence by repressing transcriptional activators of virulence gene expression [66, 67]. ANR family proteins also modulate the expression and DNA-binding properties of the global silencer H-NS, resulting in differential expression of both virulence and nonvirulence-associated genes [68, 69]. However, as a relatively new protein family, more work is needed to determine the distribution of ANR genes among vertically and horizontally acquired regions of the genome, and how these global repressors might benefit the horizontally acquired elements that encode them, like EcCIMP1. Although we were not able to detect transfer of EcCIMP1 to pathogens like *C. rodentium* or EPEC by Kapi1 (data not shown), ANR-encoding PICIs from the commensal microbiome could provide a protective effect if transferred into invading diarrheal pathogens, where they may reduce virulence.

Although EcCIMP1 does not drastically influence bacterial growth (Supplemental Fig. S2), it bolsters the fitness of Kapi1 lysogens in coculture by contributing to superinfection immunity against Kapi1 (Fig. 4AD). Using the Prokaryotic Antiviral Defense Locator (PADLOC) web server [70] and DefenseFinder [71, 72], we did not find any previously characterized defense systems in the EcCIMP1 genome, perhaps pointing to a novel defense mechanism. Because the presence of EcCIMP1 or Kapi1 alone does not confer full protection against Kapi1 (Fig. 4D), it is likely that a more complex interaction between both EcCIMP1- and Kapi1-encoded factors is required for full superinfection immunity. Phage satellites commonly restrict both helper [73] and nonhelper phages [74, 75], which benefits the bacterial host by limiting the spread of infectious virions through the population. However, our results differ from those previously described, as we only observe restriction of Kapi1 upon superinfection; prophage induction and lytic replication of Kapi1 are not restricted by EcCIMP1 (Fig. 4B, C) as it is in other PICIs [73], and Kapi1 infection of nonlysogens is not impacted by EcCIMP1 (Fig. 4D). Considering the ecology of these genetic elements and their host bacterium in their natural environment (the GI tract), it could be hypothesized that EcCIMP1-mediated superinfection immunity helps to maintain lysogeny at a population level. Piggyback-the-Winner dynamics [76] predict that lysogeny is favored over lytic replication in the GI environment [77]. In a mixed population, the unique qualities of EcCIMP1-mediated superinfection immunity would still allow for lysogenization of susceptible lineages by Kapi1, while simultaneously protecting already established lysogens from potentially lethal superinfections. In theory, this could benefit all elements involved; the host bacterium gains a prophage (increasing genetic diversity and contributing to fitness [12]) and is protected from superinfection. The phage benefits by Piggybacking-the-Winner, replicating primarily through lysogeny [12, 76]. The satellite similarly benefits by Piggybacking off the success of Kapi1 lysogens and maintains a mutualistic association with its helper phage.

One particularly unique feature of EcCIMP1 is that it has two genes annotated as AlpA family transcriptional regulators, but with differing functions. The first, *alpA*, is encoded in the conserved location just downstream of the integrase (Fig. 1A) [35, 36] and appears to be the canonical AlpA master regulator of the PICI cycle (Fig. 2) [35]. The second, *alpA_1*, is located at the opposite end of the PICI and in the opposite orientation to most other genes on the PICI (Fig. 1A). Deletion of *alpA_1* does not impact excision or packaging of EcCIMP1 (Fig. 2A and C), indicating that it is not required for the core PICI lifecycle. We noted that EcCIMP1 *alpA_1* shares sequence similarity with Kapi1 excisionase, *xis* (Fig. 5C, Supplemental Table S6), leading us to investigate whether *alpA_1* might be involved in helper phage excision. Deletion of EcCIMP1 *alpA_1* does not impact WT Kapi1 but does worsen the replication defect of a Kapi1 ∆*xis* mutant (Fig. 6A), indicating that *alpA_1* can likely stand-in for Kapi1 *xis* when it is absent. Although this may seem like a somewhat synthetic phenotype, prophages are subject to purifying selection by their bacterial hosts [78], resulting in heavily degraded prophage sequences littered throughout bacterial genomes. Because this selection most often results in the loss of lytic genes [79], EcCIMP1 may encode *alpA_1* as an “insurance policy” in case it encounters a potential helper prophage that has lost the ability to excise. In our review of the literature, we were only able to find one report of trans-acting excisionases between multiple pathogenicity islands in *V. cholerae* [80]. Here, we demonstrate excisionase crosstalk between a prophage and a PICI, supporting other recent studies suggesting that PICIs may not always be detrimental to their helper phages [9, 36, 75].

While investigating how EcCIMP1 supports helper phage excision, we noted that Kapi1 lysogens lacking both excisionase and integrase were still capable of canonical excision and subsequent lytic replication, although at a reduced frequency relative to WT (Fig. 6C and D). In our review of the literature, we have not found any reports of prophages still being able to excise and replicate through the lytic cycle in the absence of their integrase or excisionase; on the contrary, many studies utilizing *xis* and/or *int* deletions have displayed the expected cryptic prophage phenotypes [81, 82]. Although our study did not explore the molecular details of this phenotype, we have several potential hypotheses: (i) redundancy in the Kapi1 site-specific recombination (SSR) unit (i.e. other integrases and/or excisionases that are not currently annotated on the Kapi1 genome). (ii) Although we have experimental evidence that Kapi1 integrates into the bacterial chromosome [12], if a portion of the population enters a pseudolysogenic state similar to its close relative P22 [83, 84], pseudolysogens could presumably complete their lytic cycle in the absence of a functional SSR unit. (iii) Complementation via other SSR units from cohabiting MGEs, as we observed for excisionases *alpA_1* (Fig. 6A) and *alpA_5* (Fig. 7A)*. alpA_5* is encoded on a predicted 35 kb genomic island (Island 19, Supplemental Table S6), which we hypothesize is an integrative and conjugative element (ICE) due to the presence of genes involved in plasmid replication and conjugation (*repL, traYD, mobQ*), as well as a type IV pilus major pilin, and pilin tip adhesin. This classification once again underscores the novelty of our findings; excisionases from multiple different classes of MGEs, in this case a PICI and a putative ICE, can support excision of yet another class of element, a bacteriophage.

Although PICIs were traditionally viewed as phage parasites that often transmit bacterial virulence factors, recent work has started to challenge these conceptions [9, 36, 75]. Our characterization of EcCIMP1 supports the idea that PICIs are not always detrimental to their helper phages; in fact, EcCIMP1 encodes an accessory excisionase that can promote lytic replication of its helper phage Kapi1. The accessory genetic content of EcCIMP1 also reveals a gene that was bioinformatically predicted to be a virulence factor, but which actually silences virulence gene expression. Our work highlights the diversity of interactions between MGEs coinfecting a single cell and how these interactions might subsequently impact bacterial virulence and fitness. Further characterization of this extensive network of interactions will have important implications on the ways that we view and classify MGEs, as their characteristics are influenced not only by their own genetic content but also that of coresident MGEs.

## Supplementary Material

Supplementary_Material_wrae258

Supplemental_Table_S1_wrae258

Supplemental_Table_S3_wrae258

Supplemental_Table_S4_wrae258

Supplemental_Table_S5_wrae258

## Data Availability

EcCIMP1 genome sequence and annotation is deposited at NCBI Genbank under accession number OR778291 and is available at https://www.ncbi.nlm.nih.gov/nuccore/OR778291.1.
