# Supplementary material for: Escherichia coli phage-inducible chromosomal island aids helper phage replication and represses the locus of enterocyte effacement pathogenicity island
Source: ISME J. 2025 Jan 5;19(1):wrae258. doi: 10.1093/ismejo/wrae258 (PMC11773190; doi:10.1093/ismejo/wrae258)
Supplement: Supplementary_Material_wrae258 [file supplementary_material_wrae258.pdf]

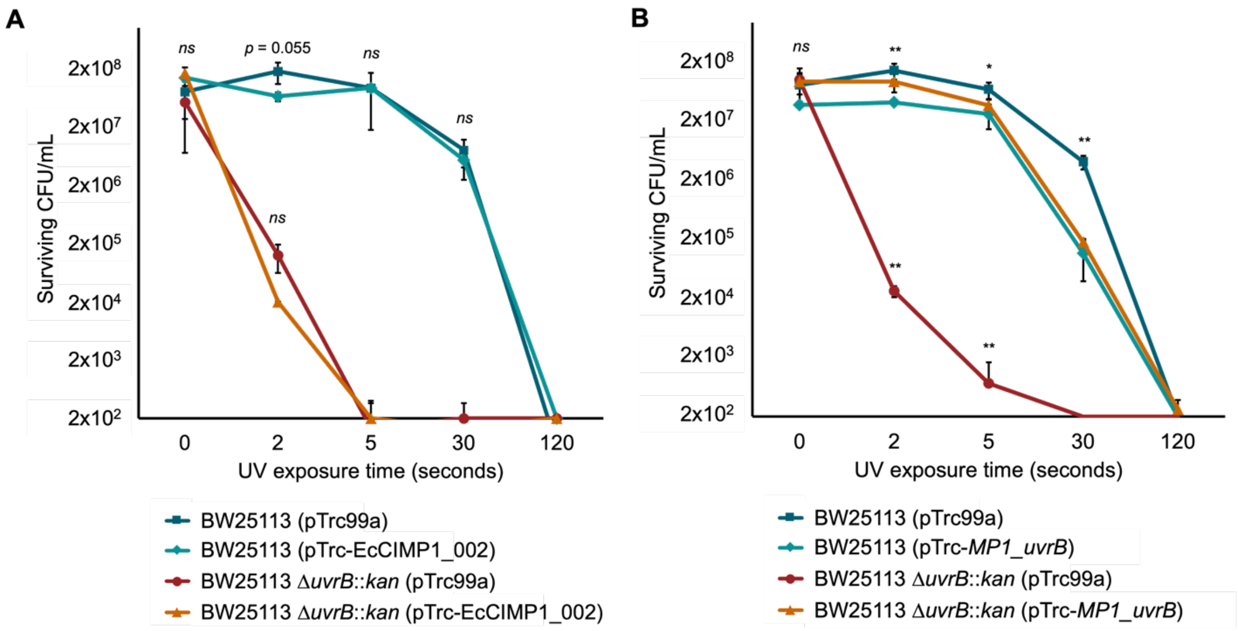

Supplemental Figure S1. EcCIMP1_002 does not complement chromosomal *uvrB.* Overnight cultures were sub-cultured 1:50 into 2 mL LB+Amp for 1 h. 0.1mM IPTG was added, then cultures were grown for an additional 1 h. Cultures were serially diluted in LB+Amp+IPTG, then 5 μL was spotted onto LB+Amp+IPTG plates. Once dry, all plates were moved into a biosafety cabinet and the lids were removed. The biosafety cabinet UV lamp was switched on, and petri plate lids were replaced at the indicated time points. Plates were then incubated overnight, and surviving colonies enumerated the next morning. The UV lamp was warmed up for 30 minutes before each experiment. The mean of three independent samples is plotted, with the standard deviation shown as error bars. Statistical significance was determined using a t-test comparing the WT strains carrying each vector at each time point, and comparing the Δ*uvrB::kan* strains carrying each vector at each time point (* $p \leq 0.05$, ** $p \leq 0.01$). Values below the limit of detection (200 CFU/mL, the origin of the graph) were excluded from statistical analysis. (A) Strains overexpressing EcCIMP1_002. (B) Control experiment with strains overexpressing MP1 chromosomal *uvrB.*

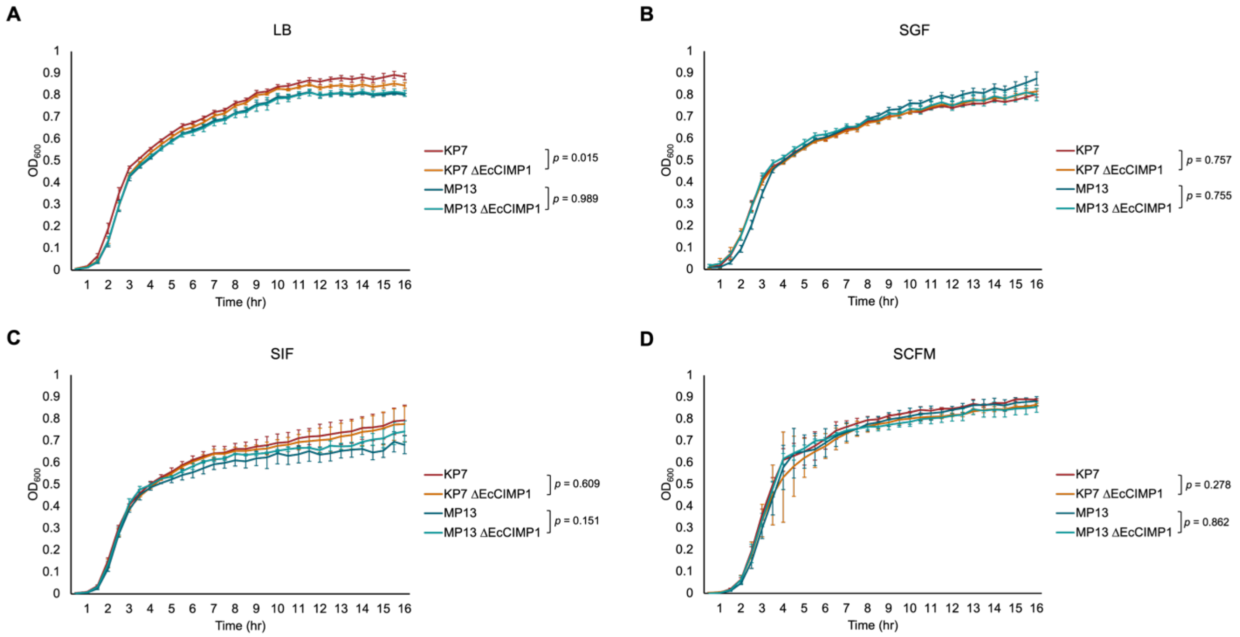

Supplemental Figure S2. EcCIMP1 does not alter bacterial growth kinetics. Bacterial growth curves were performed in LB, simulated gastric (SGF) [1,2], intestinal (SIF) [1,2], or colonic (SCFM) [3,4] fluids as described in [1], except that OD_{600} adjustments were performed in LB. The mean of three independent samples is plotted, with the standard deviation shown as error bars. Statistical significance was determined by first calculating the empirical area under the curve (AUC) for each independent sample using Growthcurver [5], then performing a t-test on the AUC values.

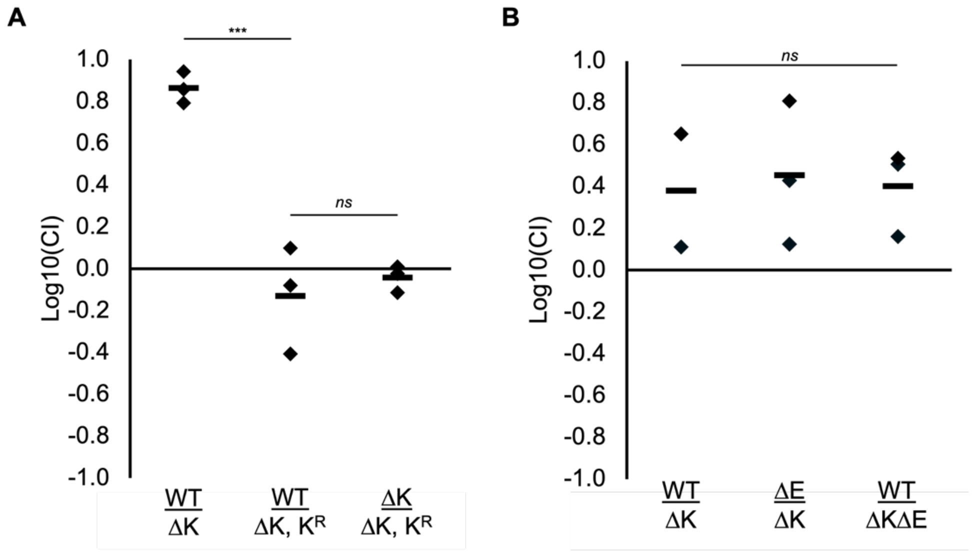

Supplemental Figure S3. Competition outcomes between Kapi1 lysogens and non-lysogens are driven by phage-mediated lysis of susceptible competitors. Bacterial competition outcomes after 48 h co-culture in LB. WT MP1 carries both Kapi1 and EcCIMP1, ΔK indicates deletion of the Kapi1 prophage, (A) K^R indicates Kapi1-resistant mutant (truncated Wzy [1]), and (B) ΔE indicates deletion of EcCIMP1. The strain designated as the numerator is tagged with *gfp*, and the denominator is tagged with *mcherry*. The competitive index (CI) was calculated as ((*gfp* CFU/mL / *mcherry* CFU/mL) / (*gfp* CFU/mL input / *mcherry* CFU/mL input)). Three independent samples are plotted as diamonds, with the mean value shown as a horizontal bar. Statistical significance was determined with t-tests (*** $p \leq 0.001$).

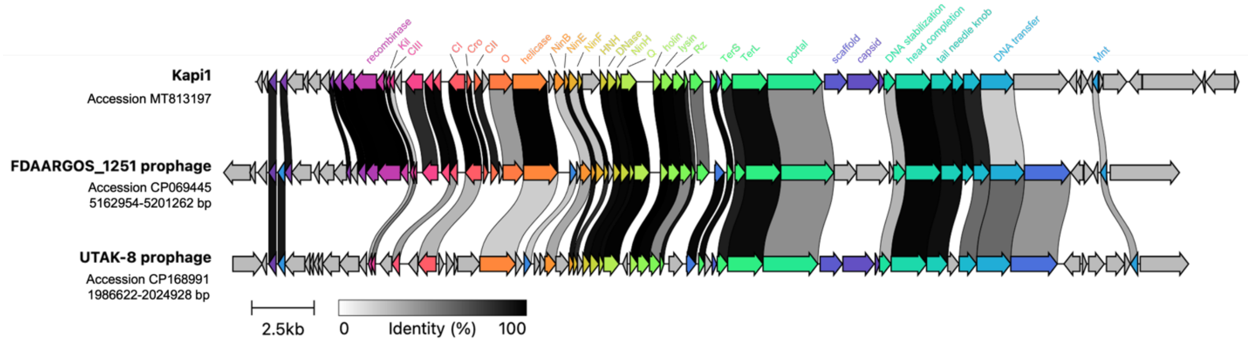

Supplemental Figure S4. Kapi1-like prophages identified in two strains of *Escherichia coli* which carry EcCIMP1. BLASTn [6] was used to search for Kapi1-like regions in the genomes of all 21 EcCIMP1-containing strains (Supplemental Table S4). Only FDAARGOS_1251 and UTAK-8 had regions with broad sequence similarity across the entire Kapi1 genome. These regions of similarity were extracted and aligned with Kapi1 using clinker [7].

```
Xis (WPV06447.1)      --------MQHELQPDSLVDLKFIMADTGFGKTFIYDRIKSGDLPKAKVIHGRA-RWLYRDHCEFKNKLLSRANG---
AlpA_5 (WPV06873.1)   ------MKENSENSVKRFIRVPDVLNRVGFSRTTLYERIKEGNFPDRVKIGPRCVAFVESEIDEWIEATIRNSRQSAA
AlpA_1 (WUR10157.1)   ---------------MRLIKLSEVMLRTGFKKTAIYSWVKTGTFPQPVKIG-RSARWSLEEVEAWIQNKLDSRAGNQ-
AlpA (WPV04227.1)     -----------MANYNSLIRLSEVQRRTGYSKAWIYRLISQGRFPKQVKIGSRAIAFVESEIDEWIEKCILESRDEVA
AlpA_2 (WPV04282.1)   MLTTTSHDSVFLRADNSLIDMNYITSFTGMTDKWFYKLISEGHFPKPIKLG-RSSRWYKREVEQWK-----------
AlpA_3 (WPV04288.1)   ------MTTPVSLMDDQMVDMTFITQLTGLTDKWFYKLIKDGAFPAPIKLG-RSSRWLKSEVEAWLQARIAQSRP---
AlpA_4 (WPV04633.1)   ------MTTPVSLMDDQMVDMAFITQLTGLTDKWFYKLIRDGAFPAPIKLG-RSSRWLKSEVEAWLQARIAQSRP---
```

Supplemental Figure S5. MUSCLE alignment between Kapi1 Xis-like proteins found in the MP1 genome. Multiple sequence alignment was performed using MUSCLE at EMBL-EBI [8]. NCBI protein IDs are provided in brackets next to each protein name. Residues conserved between all seven sequences are bolded and highlighted in orange. Residues uniquely conserved between Kapi1 Xis and the proteins with functional excisionase activity (AlpA_1 and AlpA_5) are bolded, underlined and highlighted in red. Residues uniquely conserved between the Xis and the proteins which did not support Kapi1 excision are bolded and highlighted in dark blue (AlpA, AlpA_2, AlpA_3, and AlpA_4).

Supplemental Table S2. Plasmids used in this study.

Plasmid Name	Description	Source
pRE112	Suicide vector for allelic exchange	[9]
pNLP10	Low copy number vector containing promoterless *luxCDABE* operon	[10]
pNLP10 P*alpA-lux*	EcCIMP1 *alpA* promoter region (-449 to +50 relative to start codon) cloned into MCS, drives expression of *luxCDABE*	This study
pNLP10 P*CI-lux*	Kapi1 *CI* promoter region (-86 to +13 relative to start codon) cloned into MCS, drives expression of *luxCDABE*	[1]
pNLP10 P*ler-lux*	*Citrobacter rodentium* DBS100 *ler* promoter region (-397 to +49 relative to start codon) cloned into MCS, drives expression of *luxCDABE*	[3]
pTrc99A	Expression vector, IPTG-inducible from *trc* promoter	[11]
pTrc99A *alpA*	Coding sequence of EcCIMP1 *alpA* cloned into MCS, expressed from P*trc*	This study
pTrc99A *EcCIMP1_002*	Coding sequence of *EcCIMP1_002* cloned into MCS, expressed from P*trc*	This study
pTrc99A *MP1_uvrB*	Coding sequence of MP1 *uvrB* cloned into MCS, expressed from P*trc*	This study
pTrc99A *EcCIMP1_025*	Coding sequence of *EcCIMP1_025* cloned into MCS, expressed from P*trc*	This study
pTrc99A *EPEC_perC*	Coding sequence of enteropathogenic *E. coli* E2348/69 *perC* from pEAF plasmid cloned into MCS, expressed from P*trc*	This study
pTrc99A *alpA_2*	Coding sequence of MP1 *alpA_2* cloned into MCS, expressed from P*trc*	This study
pTrc99A *alpA_3*	Coding sequence of MP1 *alpA_3* cloned into MCS, expressed from P*trc*	This study
pTrc99A *alpA_4*	Coding sequence of MP1 *alpA_4* cloned into MCS, expressed from P*trc*	This study
pTrc99A *alpA_5*	Coding sequence of MP1 *alpA_5* cloned into MCS, expressed from P*trc*	This study
pTrc99A *intB_2*	Coding sequence of MP1 *intB_2* cloned into MCS, expressed from P*trc*	This study
pTrc99A *intB_3*	Coding sequence of MP1 *intB_3* cloned into MCS, expressed from P*trc*	This study
pTrc99A *intZ*	Coding sequence of MP1 *intZ* cloned into MCS, expressed from P*trc*	This study

Supplemental Table S6. BLASTp results when Kapi1 Xis amino acid sequence (accession QOC59519) is searched against all protein sequences from the MP1 genome (accession CP109039).

MP1 locus_tag	Product	Colloquial gene name	Query Cover	E- value	% Identity	Genomic Location (locus_tag range)
SID94_17316	AlpA family transcriptional regulator	*alpA_1*	83%	7e-08	42.86%	EcCIMP1 (SID94_17295-SID94_17420)
SID94_17735	AlpA family transcriptional regulator	*alpA_2*	75%	3e-04	30.00%	GIPSy: Putative island 13 (SID94_17685-SID94_17740)
SID94_17775	AlpA family transcriptional regulator	*alpA_3*	69%	8e-04	34.78%	PHASTER: Putative prophage 6 (SID94_17770-SID94_18020)
SID94_17415	AlpA family transcriptional regulator	*alpA*	59%	0.001	38.46%	EcCIMP1 (SID94_17295-SID94_17420)
SID94_19740	AlpA family transcriptional regulator	*alpA_4*	69%	0.002	32.61%	GIPSy: Putative island 16 (SID94_19730-SID94_19790) GIPSy: Putative island 24 (SID94_19735-SID94_19865)
SID94_09055	AlpA family transcriptional regulator	*alpA_5*	33%	0.013	40.91%	GIPSy: Putative island 19 (SID94_09030-SID94_09235)

Supplemental Table S7. BLASTp results when Kapi1 Int amino acid sequence (accession QOC59585) is searched against all protein sequences from the MP1 genome (accession CP109039).

MP1 locus_tag	Product	Colloquial gene name	Query Cover	E- value	% Identity	Genomic Location (locus_tag range)
SID94_17420	tyrosine-type recombinase / integrase	*intS_1*	99%	2e-137	49.36%	EcCIMP1 (SID94_17295-SID94_17420)
SID94_19955	integrase arm-type DNA-binding domain-containing protein	*intB_3*	99%	3e-71	33.42%	GIPSy: Putative island 25 (SID94_19905-SID94_19955)
SID94_09030	integrase arm-type DNA-binding domain-containing protein	*intB_2*	98%	6e-69	30.10%	GIPSy: Putative island 19 (SID94_09030-SID94_09235)
SID94_09235	integrase arm-type DNA-binding domain-containing protein	*intZ*	85%	9e-28	30.68%	PHASTER: Putative prophage 2 (SID94_09180-SID94_09235) GIPSy: Putative island 19 (SID94_09030-SID94_09235)
SID94_17290	Arm DNA-binding domain-containing protein; pseudogene (missing C-terminus)	*intS_2*	26%	3e-23	44.04%	Unclassified putative genomic island adjacent to EcCIMP1 (SID94_17290-SID94_17265)
SID94_09215	recombinase; pseudogene (missing N-terminus)	*intB_1*	17%	2e-14	44.44%	PHASTER: Putative prophage 2 (SID94_09180-SID94_09235) GIPSy: Putative island 7 (SID94_09195-SID94_09230) GIPSy: Putative island 19 (SID94_09030-SID94_09235)

Supplemental Tables S1, S3-S5 are available as separate Excel files:

- Supplemental Table S1. Bacterial strains used in this study.
- Supplemental Table S3. Primers used in this study.
- Supplemental Table S4. Top 100 BLASTn results when EcCIMP1 is searched against the NCBI core_nt database.
- Supplemental Table S5. Nucleotide sequences of Gram-negative PICIs and PLEs used for phylogenetic analysis.

Supplemental References:

1. Pick K, Ju T, Willing BP *et al.* Isolation and characterization of a novel temperate escherichia coli bacteriophage, Kapi1, which modifies the O-antigen and contributes to the competitiveness of its host during colonization of the murine gastrointestinal tract. *mBio* 2022;**13**:e02085-21.

2. Millette M, Nguyen A, Amine KM *et al.* Gastrointestinal survival of bacteria in commercial probiotic products. *International Journal of Probiotics and Prebiotics* 2013;**8**:149–56.

3. Gilliland A, Gavino C, Gruenheid S *et al.* Simulated colonic fluid replicates the in vivo growth capabilities of citrobacter rodentium cpxRA mutants and uncovers additive effects of cpx-regulated genes on fitness. *Infection and Immunity* 2022;**90**:e00314-22.

4. Beumer RR, de Vries J, Rombouts FM. Campylobacter jejuni non-culturable coccoid cells. *International Journal of Food Microbiology* 1992;**15**:153–63.

5. Sprouffske K, Wagner A. Growthcurver: an R package for obtaining interpretable metrics from microbial growth curves. *BMC Bioinformatics* 2016;**17**:172.

6. Altschul SF, Gish W, Miller W *et al.* Basic local alignment search tool. *Journal of Molecular Biology* 1990;**215**:403–10.

7. Gilchrist CLM, Chooi Y-H. Clinker & clustermap.js: Automatic generation of gene cluster comparison figures. *Bioinformatics* 2021;**37**:2473–5.

8. Madeira F, Pearce M, Tivey ARN *et al.* Search and sequence analysis tools services from EMBL-EBI in 2022. *Nucleic Acids Res* 2022;**50**:W276–9.

9. Edwards RA, Keller LH, Schifferli DM. Improved allelic exchange vectors and their use to analyze 987P fimbria gene expression. *Gene* 1998;**207**:149–57.

10. Price NL, Raivio TL. Characterization of the Cpx regulon in Escherichia coli strain MC4100. *Journal of Bacteriology* 2009;**191**:1798–815.

11. Amann E, Ochs B, Abel KJ. Tightly regulated tac promoter vectors useful for the expression of unfused and fused proteins in Escherichia coli. *Gene* 1988;**69**:301–15.
